# Vision-Based Finger Detection, Tracking, and Event Identification Techniques for Multi-Touch Sensing and Display Systems

**DOI:** 10.3390/s110706868

**Published:** 2011-07-01

**Authors:** Yen-Lin Chen, Wen-Yew Liang, Chuan-Yen Chiang, Tung-Ju Hsieh, Da-Cheng Lee, Shyan-Ming Yuan, Yang-Lang Chang

**Affiliations:** 1 Department of Computer Science and Information Engineering, National Taipei University of Technology, 1, Sec. 3, Chung-hsiao E. Rd., Taipei 10608, Taiwan; E-Mails: ylchen@csie.ntut.edu.tw (Y.-L.C.); wyliang@csie.ntut.edu.tw (W.-Y.L.); tjh@csie.ntut.edu.tw (T.-J.H.); abel0421@gmail.com (D.-C.L.); 2 Department of Computer Science, National Chiao Tung University, 1001 University Road, Hsinchu 30050, Taiwan; E-Mails: gmuooo@gmail.com (C.-Y.C.); smyuan@gmail.com (S.-M.Y.); 3 Department of Electrical Engineering, National Taipei University of Technology, 1, Sec. 3, Chung-hsiao E. Rd., Taipei 10608, Taiwan

**Keywords:** multi-touch sensing, computer vision, finger detection, finger tracking, multi-touch event identification

## Abstract

This study presents efficient vision-based finger detection, tracking, and event identification techniques and a low-cost hardware framework for multi-touch sensing and display applications. The proposed approach uses a fast bright-blob segmentation process based on automatic multilevel histogram thresholding to extract the pixels of touch blobs obtained from scattered infrared lights captured by a video camera. The advantage of this automatic multilevel thresholding approach is its robustness and adaptability when dealing with various ambient lighting conditions and spurious infrared noises. To extract the connected components of these touch blobs, a connected-component analysis procedure is applied to the bright pixels acquired by the previous stage. After extracting the touch blobs from each of the captured image frames, a blob tracking and event recognition process analyzes the spatial and temporal information of these touch blobs from consecutive frames to determine the possible touch events and actions performed by users. This process also refines the detection results and corrects for errors and occlusions caused by noise and errors during the blob extraction process. The proposed blob tracking and touch event recognition process includes two phases. First, the phase of blob tracking associates the motion correspondence of blobs in succeeding frames by analyzing their spatial and temporal features. The touch event recognition process can identify meaningful touch events based on the motion information of touch blobs, such as finger moving, rotating, pressing, hovering, and clicking actions. Experimental results demonstrate that the proposed vision-based finger detection, tracking, and event identification system is feasible and effective for multi-touch sensing applications in various operational environments and conditions.

## 1. Introduction

Multi-touch sensing systems have attracted a lot of attention in current human-computer interaction applications [1]. The first prototype multi-touch sensing system was developed at the University of Toronto at 1982 [2]. Bell Labs then designed a touch sensing and display system for graphical user interface applications [3] in 1984. Many well-known commercial products with multi-touch technologies have recently become in everyday life, such as the Apple iPhone [4], Apple Macbook Air [5], and Microsoft Surface Computer [6]. A multi-touch interface provides much more flexibility and convenience than traditional interfaces such as keyboards and mouses by allowing user to directly and intuitively interact with digital content and media through hand and finger actions on the display surfaces.

Some consumer systems, such as the touch screens on electronic check-out machines, use resistive surfaces for touch sensing. These resistance-based systems are cheap to implement, but cannot provide sufficient sensing accuracy and clarity to support more sophisticated user operations and commands. Systems based on capacitive technologies can provide more accurate sensing results using multiple touch contacts on the screen [7–9]. However, systems based on capacitive sensing require some specific facilities, such as specified receivers for detecting the signals emitted from the transmitter under the display surface. This significantly limits the possible movements and operations of many user applications. Infrared LED based systems [10–12] use sets of pairing transmitters and receivers of infrared LEDs to construct a two dimensional sensing array on the back of a screen. This type of system can sense touch contact through blocked infrared lights when a user’s finger touches a given cross point of a horizontal path and a vertical path of infrared lights in the sensing array. Nevertheless, the touch sensing accuracy of infrared LED based systems relies heavily on the number and density of transmitters and receivers, and the structural arrangement of the sensing array. Thus, their applicability and flexibility are limited when large display screens must accommodate more users and more simultaneous touch operations.

Due to the falling costs and growing power of computers, technologies based on computer vision are becoming popular solutions for many object detection, tracking, and analysis applications in real-life environments, such as the detection and recognition of people, vehicles, lanes, animals, obstacles, *etc*… [13–20]. For multi-touch sensing applications, hands and fingers are the salient objects captured from the cameras in the computer vision problems. Thus, camera-based techniques have attracted a lot of attention in multi-touch sensing applications [21–30]. Several camera-based systems apply computer vision techniques to detect and track finger locations via overhead-mounted cameras [21–24]. Letessier and Berard [21] presented a single overhead camera system to capture finger positions on a display surface. The single overhead camera approach has obvious difficulties in accurately detecting finger contacts on the display surface. Systems based on paired overhead cameras [22,23] use stereo vision and depth features to provide more precise detected finger contacts with the display surface. However, these systems still suffer significant self-occlusion problems when multiple fingers are simultaneously pressed on the surface. These multiple camera systems also require additional costs in camera calibration before use. The PlayAnywhere system in [24] adopts an infrared camera and uses the shape of a finger shadow to refine the touch detection results. However, when multiple finger contacts are pressed on the surface and the pointing directions of these fingers are not perpendicular to the direction of the infrared light source, the shadow shape features become unreliable due to occlusions and lighting effects. Chung *et al*.’s MirrorTrack system [25,26] uses three cameras side-mounted parallel to the glossy display surface to capture finger contacts on the surface in three different orientations. This system provides more accurate detection results for finger contact points. However, due to the restricted capturing fields of the side-mounted cameras, more cameras are required to capture sufficient image features of the hands of the users in the surface area. Thus, the costs of multiple cameras and the complexity of calibrating multiple cameras limit the applicability and convenience of side-mounted camera based systems.

**Figure 1.**
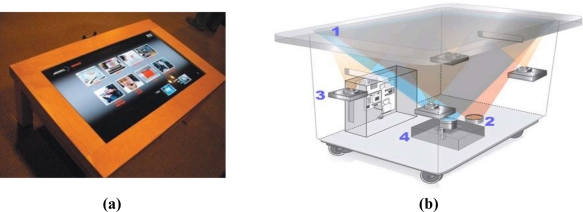
**(a)** A Microsoft Surface Computer. **(b)** The structure of Microsoft Surface (1 is the transparent panel, 2 is the infrared source, 3 is a infrared camera, 4 is the projector).

Researchers have recently develop rear-mounted camera based systems for consumer multi-touch sensing applications [6,27,28]. The Diffused Illumination (DI) technique adopted in Microsoft’s Surface computer [6] product uses one infrared emitter, four infrared cameras, and a projector (Figure 1). This system emits infrared lights onto a screen, and once the user touches the screen, the four cameras capture the reflected infrared lights and produce bright blobs. These bright blobs are the small spots of reflected lights formed by the users’ touch contacts, and can be detected and processed via some image processing techniques. The HoloWall system [27] uses a glass wall combined with a rear-projection sheet to capture the scattered infrared lights caused by finger touches. This technique is more inexpensive and easy to implement. However, when finger touches occur, the infrared lights may be scattered wide, making it difficult for the camera to capture sufficient lights to identify the touch blobs of interest. Moreover, this technique is sensitive to ambient lighting effects. Han [28] proposed a Frustrated Total Internal Reflection (FTIR) technique, which is inexpensive and easy to implement. The FTIR technique uses a specialized acrid panel as the display surface, an infrared emitter, a projector, and a video camera. The FTIR technique uses infrared LEDs to emit infrared lights into the acrid display panel from one side to another side. The infrared lights are totally reflected within the acrid panel until finger touches scatter the lights. These scattered lights can then be captured by a video camera mounted under or behind the acrid display panel and imaged as bright blobs. These bright blobs can also be detected and recognized as finger touches via some image processing techniques to obtain possible interactive actions of the users.

The FTIR technique offers great flexibility in simultaneously detecting a large number of multiple touch contacts with high spatial and temporal frequencies. Other camera-based systems, such as overhead-camera-based systems, are unable to detect and process multiple nearby touches or provide some advanced interacting operations. However, because each touch contact detected by the FTIR appears as an independent event, post-processing is necessary to determine whether a set of touch contacts detected at different time belong to the same user’s touch trajectory. As the number of user grows and their corresponding touch actions increase, the determination of user operations from a large number of individual touch detections becomes increasingly difficult. The touch detection accuracy of the FTIR technique may be affected by spurious infrared noises under poor lighting conditions.

To overcome the above-mentioned problems of the FTIR technique, this study presents efficient vision-based touch finger detection, tracking, and event identification techniques and a low-cost hardware framework for multi-touch sensing and display applications. First, a fast bright-blob segmentation process based on automatic multilevel histogram thresholding extracts the pixels of touch blobs formed by scattered infrared lights in the image sequences captured by a video camera. The advantage of this automatic multilevel thresholding approach is its robustness and adaptability when dealing with various ambient lighting conditions and spurious infrared noises. A connected-component analysis procedure is then applied to the bright pixels obtained by the previous stage to extract the connected-components of these touch blobs. Given the touch blobs extracted from each of the captured frames, a blob tracking and event recognition process analyzes the spatial and temporal information of these touch blobs from consecutive frames to determine possible touch events. This process also refines the detection results and corrects for errors and occlusions caused by noise and errors during the blob extraction processes. The proposed blob tracking and touch event recognition process includes two phases. The blob tracking phase first associates the motion correspondence of blobs in succeeding frames by analyzing their spatial and temporal features. The touch event recognition phase then identifies meaningful touch events and gestures from the motion information of touch blobs (such as finger moving, clicking, and zooming actions). The resulting touch event and gestures represent various interactive commands for controlling and manipulating objects on the display interface. Experimental results demonstrate that the proposed vision-based finger detection, tracking, and event identification system is feasible and effective for multi-touch sensing applications in various operational environments and illumination conditions.

## 2. Hardware Structure Design

**Figure 2.**
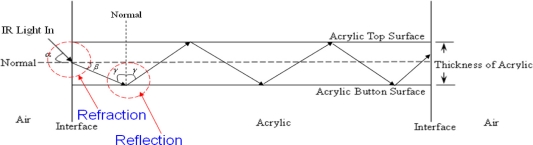
Total internal reflection in an acrylic board.

**Figure 3.**
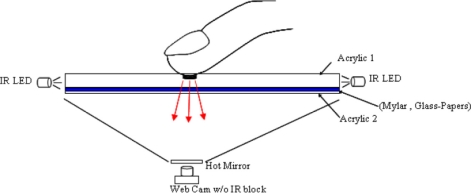
The structure of the proposed multi-touch sensing interface.

Frustrated total internal refection (FTIR) [28] is based on the phenomenon of the total internal reflection of infrared lights within an acrylic board (Figure 2). Figure 3 illustrates the structure of the proposed multi-touch sensing interface, showing that the infrared lights are emitted into the inner layer of a transparent acrylic board with a proper angle via the side-mounted infrared LEDs. Without exterior interference, the lights pass through the inner layer of the board due to total reflection phenomenon. When the top side of the surface is pressed by an object with a relatively high refractive rate, such as a finger, some of the infrared light at the contact point is reflected and diffused to the bottom of the board. The diffused infrared lights can be captured by a video camera mounted below the acrylic board and detected as bright blobs. These bright blobs can then be processed using image segmentation and recognition techniques to locate and analyze their appearance and motion features, and generate interactive commands for various applications.

**Figure 4.**
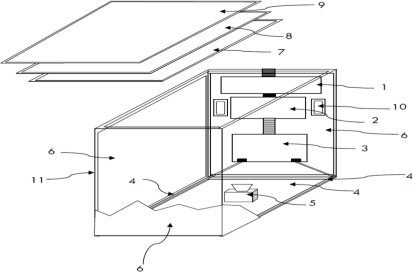
The device design and components of the proposed multi-touch sensing and display system. 1: main circuit board module; 2: AD/DA module; 3: backlight circuit module; 4: backlights; 5: low-cost web-camera; 6: aluminum foil; 7: LCD panel; 8: cellophane paper; 9: acrylic board; 10: speakers; 11: device case.

This study presents a low-cost solution for multi-touch sensing and display based on the FTIR concept. Figure 4 depicts the design framework of the proposed multi-touch sensing and display device. A conventional LCD displayer serves as the display interface. Multi-touch sensing modules are placed in front of the LCD displayer for sensing and obtaining the user finger touching blobs at the input interface. As Figure 4 illustrates, the LCD displayer is split into the following parts and installed in the device case: the main circuit board module (Figure 4:1), the AD/DA (analog to digital/digital to analog) module (Figure 4:2), the backlight circuit module (Figure 4:3), and backlights (Figure 4:4). The LCD panel (Figure 4:7) is installed on top of the case (Figure 4:11). To capture the reflected lights of the touch fingers, a low-cost web-camera (Figure 4:5) serves as the image capturing interface. To capture the reflected infrared lights efficiently, the IR-block filter (infrared filter) of the camera is removed and replaced by a band-pass filter so that only infrared lights can be passed and captured. To smooth the backlight in the case, aluminum foil (Figure 4:6) is attached to the inner surface of the device case, and some cellophane paper (Figure 4:8) is placed under the acrylic board to improve the smoothness of the backlights. Finally, an acrylic board (Figure 4:9) and infrared LEDs are installed above the LCD panel (Figure 4:7) to capture multi-touch inputs. The remaining components, *i.e.*, the speakers associated with the original LCD displayer, are also installed in the case (Figure 4:10). As a result, the total cost of the prototype system of the proposed multi-touch sensing and display device is estimated to be less than USD$ 300.

## 3. Vision-Based Multi-Touch Sensing Technique

Using the finger touching blobs obtained by the proposed multi-touch sensing interface, image segmentation, object extraction and tracking, and event identification techniques must locate and analyze their appearance and motion features to determine possible interactive computer commands. This section presents efficient vision-based finger detection, tracking, and event identification techniques to identify the desired user commands effectively.

### 3.1. Touching Blob Extraction

**Figure 5.**
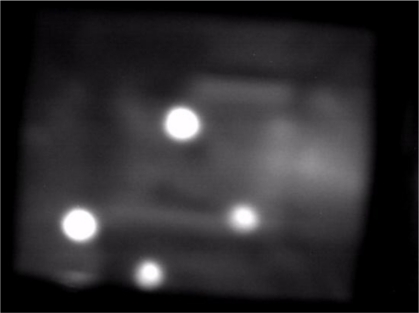
Example of the captured image from the proposed sensing interface.

The first step in detecting and extracting the touched fingers captured from the camera of the multi-touch sensing system is to segment the salient features of moving blobs from captured image sequences. Figure 5 shows a sample of the captured image from the proposed sensing interface. Performing blob detection and recognition for multi-touch display applications requires an effective approach for correctly and rapidly locating and extracting the salient features of blobs under various illumination conditions on the sensing interface. This way enables the efficient extraction and segmentation of the object regions of touching fingers. Therefore, this section presents a fast bright-object segmentation process based on automatic multilevel histogram thresholding. The proposed method extracts the object pixels of finger touching blobs from captured image sequences.

The first task of the image-processing module in the proposed multi-touch sensing system is to extract the object pixels of finger touching blobs from the captured sensing image sequences to facilitate further rule-based analysis of the touch events and user-activated commands. To reduce the computation cost of extracting finger touching blobs, the gray intensity image, *i.e.*, the Y-channel, of the image is first extracted by performing a RGB to Y transformation. To extract finger blob objects from a given transformed gray-intensity image, finger blob pixels must be separated from other uninteresting object pixels of different illumination features. The proposed approach uses the discriminant criterion to measure separability among the decomposed images with dissimilar objects. Otsu [31] was the first to use the discriminant criterion for the bi-level image thresholding. Otsu determined the optimal threshold by maximizing the between-class variance between dark and bright regions of the image. This method is ranked as the most effective bi-level thresholding method for image segmentation [32]. Extensive research studies based on this optimal thresholding methodology have also been efficiently applied to various object segmentation and analysis applications, such as text extraction for document image analysis [33,34], biofilm image segmentation [35,36], and vehicle detection applications [20]. However, as the number of desired thresholds of different objects increases, the computational cost needed to obtain the optimal threshold values increases significantly, and the search to achieve the optimal value of the criterion function becomes particularly exhaustive.

An effective multilevel thresholding technique is needed to automatically determine the appropriate number of thresholds to extract touch blob regions from multi-touch sensing image sequences. Using the properties of discriminant analysis, our previous research presents an effective automatic multilevel thresholding technique for image segmentation [37]. This technique extends and adopts the properties of discriminant analysis to multilevel thresholding. By evaluating the separability using the discriminant criterion the number of objects, into which the image frame should be segmented, can be automatically determined to accordingly extract salient objects of interest. As a result, the pixel regions of touch blobs can be appropriately extracted from other uninteresting objects contained in the multi-touch sensing images.

The proposed touch blob segmentation process is briefly described as follows. Let *f_i_* denote the observed occurrence frequencies (histogram) of all pixels in a given captured image ***I***, with a given grayscale intensity *i*, and let *N* denote the total number of pixels in the image ***I***, and can be given by *N* = *f*_0_ + *f*_1_ + … + *f_L_*_–1_, where *L* is the number of grayscale intensities in the histogram, and represents the brightest grayscale value, *i.e.*, 255 in our vision system. Hence, the normalized probability *P_i_* of one pixel having a given grayscale intensity *i* can be denoted as:

(1) $$P_{i}=f_{i}/N,\;\text{where }P_{i}\geq 0,\sum_{i=0}^{L-1}P_{i}=1$$

If the image ***I*** consists of multiple objects, it is possible to extract pixels of bright object regions (*i.e.*, touch blobs in the sensing images) from those of other homogeneous object regions by partitioning pixels into a suitable number of classes, each of which consists of one respective homogenous object region. Thus, a multiple threshold set ***T*** comprised of *k* thresholds, *i.e.*, ***T*** = {*t*_1_,..., *t_n_*,...,*t_k_*} can be determined to segment pixels of the image ***I*** into *k*+1 homogenous pixel classes. These segmented pixel classes are represented by *C*_0_ = {0,1,..., *t*_1_} ,…, *C_n_* = {*t_n_* + 1, *t_n_* + 2,..., *t_n_*_+1_}, …, *C_k_* = {*t_k_* + 1, *t_k_* + 2,..., *L*–1}. Here, the class *C_k_* consists of pixels of bright blob objects of interest, that is, the finger touch blobs in the sensing images.

This study uses several statistical measures for performing this multilevel thresholding process. The between-class variance, denoted by *v_BC_*, an effective criterion for evaluating the results of segmentation, is utilized to measure the separability among all pixel classes. This criterion is expressed as:

(2) $$v_{BC}(\text{T})=\sum_{n=0}^{k}w_{n}{\left(\mu_{n}-\mu_{T}\right)}^{2}$$

where *w_n_* is the cumulative probability mass function of class *C_n_*, and *μ_n_* denotes the mean of pixels in class *C_n_*. The within-class variance, denoted by *v_WC_*, of all segmented classes of pixels is:

(3) $$v_{WC}(\text{T})=\sum_{n=0}^{k}w_{n}\sigma_{n}^{2}$$

The total variance *v_T_* and the overall mean *μ_T_* of pixels in the image ***I*** are:

(4) $$v_{T}=\sum_{i=0}^{L-1}{\left(i-\mu_{T}\right)}^{2}P_{i},\text{and }\mu_{T}=\sum_{i=0}^{L-1}iP_{i}$$

where *k* is the number of selected thresholds to segment pixels into *k* + 1 classes, and *σ_n_* represents the standard deviation of pixels in class *C_n_*. The statistical terms *w_n_*, *μ_n_*, and *σ_n_* of pixels in class *C_n_* are computed respectively as:

(5) $$w_{n}=\sum_{i=t_{n}+1}^{t_{n}+1}P_{i},\;\;\;\mu_{n}=\frac{\sum_{i=t_{n}+1}^{t_{n}+1}iP_{i}}{w_{n}},\;\;\text{and}\;\;\sigma_{n}^{2}=\frac{\sum_{i=t_{n}+1}^{t_{n}+1}P_{i}{\left(i-\mu_{n}\right)}^{2}}{w_{n}}$$

where a dummy threshold *t*_0_ = 0 is used to simplify the expression of equation terms.

The aforementioned statistical measures can be used as a measure of separability, among all existing pixel classes, decomposed from the original image ***I***. This study uses this concept as a criterion of automatic image segmentation, denoted by the “separability factor” — 𝒮𝒡, which is defined as:

(6) $$𝒮𝒡=v_{BC}(\text{T})/v_{T}=1-v_{WC}(\text{T})/v_{T}$$

where *v_T_* is the total variance of the grayscale values of the image ***I*** and serves as the normalization factor in this equation. The 𝒮𝒡 value reflects the separability among all existing classes, and the 𝒮𝒡 value lies within the range [0,1]. The segmentation results of homogeneous objects can be optimized by maximizing the 𝒮𝒡 value. Observation of the terms comprising *v_WC_* (***T***) indicate that if the pixels in each class are broadly spread, *i.e.*, the contribution of the class variance *σ_n_*^2^ is large, then the corresponding 𝒮𝒡 measure becomes small. Hence, when 𝒮𝒡 approaches 1.0, all classes of grayscale values decomposed from the original image ***I*** are ideally and completely separated. Previous research presents a detailed derivation of this property [37].

Using the above-mentioned statistical discriminant measure, the bright object segmentation process can be implemented by recursively segmenting homogeneous objects from the multi-touch sensing image ***I*** until the brightest objects of interest are clearly obtained, regardless of the number of existing objects and various illuminated conditions. This segmentation process is conducted by recursively thresholding the grayscale values of the image ***I*** until the 𝒮𝒡 measure is large enough (*i.e.*, 𝒮𝒡 approaches 1.0) to indicate that the appropriate discrepancy among the resulting classes of grayscale intensities is achieved. Thus, the bright objects of touch blobs are clearly segmented into a separate thresholded sensing image. Through the above mentioned properties, this objective can be reached by minimizing the total within-class variance *v_WC_* (***T***). This can be achieved by a recursive partition strategy that selects the pixel class with the maximal within-class variance contribution (*w_n_σ_n_*^2^), denoted by *C_p_*. The bi-class partition procedure, as described in [37], is performed on each determined *C_p_* into two more classes in each recursion. This partition process is recursively performed until the separability among all pixel classes becomes satisfactory, *i.e*., the 𝒮𝒡 measure approximates a sufficiently large value:

(7) $$𝒮𝒡\geq {\mathrm{Th}}_{S}$$

This study sets the value of the separability measure threshold *Th_S_* as 0.92 based on the experimental analysis in [37] to yield satisfactory object segmentation results under different illumination conditions. The resulting thresholded bright objects of interest, *i.e*., the obtained brightest pixel class *C_k_*, extracted by this recursive thresholding strategy are ensured to achieve maximum separation. This approach achieves satisfactory segmentation results for these objects using the fewest recursions. Previous research provides detailed descriptions of this multilevel thresholding technique [37].

**Figure 6.**
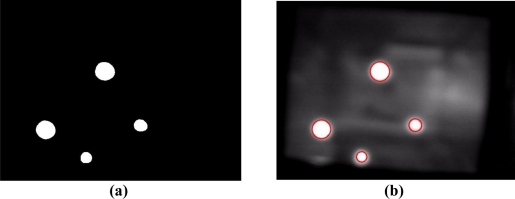
Results of performing the touching blob extraction process on the captured image in Figure 5. **(a)** Bright blobs extracted from Figure 5. **(b)** The connected-components of the bright blobs obtained from Figure 6(a).

To obtain blobs of potential touching fingers from the extracted bright object plane, a connected-component extraction process [38] is performed on the bright object plane to label and locate the connected-components of the bright blobs. Extracting the connected-components reveals the meaningful features of the location, size, and pixel distribution associated with each touching blob. Figure 6(a) shows that, after performing the touching blob extraction process, the pixels of bright blobs are efficiently segmented into thresholded object planes from the captured sensing image in Figure 5. Figure 6(b) shows the connected-components of the bright blobs obtained from Figure 6(a). The proposed blob tracking and touch event recognition approach analyzes and processes the blobs of potential touching fingers acquired in each image of video sequences captured from the vision system to identify possible touch events, as described in the following subsections.

### 3.2. Blob Tracking and Touch Event Identification

The proposed touching blob extraction method effectively obtains the blobs of the touching fingers in each image frame captured from the proposed sensing interface. In multi-touch applications, users perform various gestures by moving their fingers to issue their desired control commands. These gestures are mostly formed by the movements and actions of one, two, or more blobs of touching fingers. Since the complete features of touching gestures may not be immediately identified from single image frames, a tracking procedure for the blobs of touching fingers must be applied to analyze the information of moving blobs to recognize touching gestures from consecutive image frames. During the tracking process, the spatial-temporal information of moving blobs can be used to refine the detection results and correct the errors due to occlusions caused by noise and interference during the blob object segmentation process. The tracking information of blobs can be utilized to determine and recognize meaningful touch events, such as finger moving, rotating, pressing, hovering, and clicking actions. The proposed blob tracking and touch event recognition process includes two phases. The blob tracking phase first associates the motion of blobs in succeeding frames by analyzing their spatial and temporal features. The touch event recognition phase then identifies possible touch events based on the tracked motion information of blobs.

#### 3.2.1. Blob Tracking Process

The spatial-temporal features of the blob regions of touching fingers make it possible to progressively refine and correct the detection results of the moving blobs by associating them in sequential frames. Therefore, this study presents a tracking process for analyzing the motion features of the detected touch blobs to address the above-mentioned problems. Moreover, the tracking information of the moving blobs can be applied to the following touch event recognition process to determine possible touch events from the tracked motion information of the blobs.

When the vision system initially detects the blob of a given finger, it creates a tracker to associate this blob with those from the same finger in subsequent frames based on their contiguous spatial-temporal features. The features used in the tracking process are described and defined as follows:

1. $P_{i}^{t}$ denotes the *i^th^* detected touch blob appearing in *t^th^* frame captured by the sensing system
2. The position of the blob $P_{i}^{t}$ employed in the tracking process is represented by its centroid, and can be simply computed by:
  (8) $$P_{i}^{t}=\left(\frac{l(P_{i}^{t})+r(P_{i}^{t})}{2}\frac{t(P_{i}^{t})+b(P_{i}^{t})}{2}\right)$$
  where $l(P_{i}^{t})$, $r(P_{i}^{t})$, $t(P_{i}^{t})$, and $b(P_{i}^{t})$ denote the left, right, top, and bottom coordinates of the blob $P_{i}^{t}$, respectively
3. The tracker ${\mathrm{TP}}_{i}^{t}$ represents the trajectory of the blob $P_{i}^{t}$ that has been tracked in a set of sequential frames 1 to *t*, and is defined as:
  (9) $${\mathrm{TP}}_{i}^{t}=\langle P_{i}^{1},P_{i}^{2},\ldots ,P_{i}^{t}\rangle$$
4. Let $d_{i}^{t}$ represent the path deviation feature of a given blob $P_{i}^{t}$ at the *t^t^*^h^ frame captured by the vision system, which is:
  (10) $$d_{i}^{t}=\varphi (P_{i}^{t-2},P_{i}^{t-1},P_{i}^{t})=\varphi (\bar{P_{i}^{t-2}P_{i}^{t-1}},\bar{P_{i}^{t-1}P_{i}^{t}})$$
  where the function *φ* is the path coherence function of $P_{i}^{t}$, and the vector $\bar{P_{i}^{t-1}P_{i}^{t}}$ reveals the position deviation of $P_{i}^{t}$ from the *t* − 1*^t^*^h^ frame to the *t^t^*^h^ frame.
5. The path coherence function *φ* can then be determined by computing the deviation formula of the motion vectors $\bar{P_{i}^{t-2}P_{i}^{t-1}}$ and $\bar{P_{i}^{t-1}P_{i}^{t}}$ in two succeeding frames. The path coherence function *φ* consists of two deviation terms. The first term reveals the directional deviation formed by $\bar{P_{i}^{t-2}P_{i}^{t-1}}$ and $\bar{P_{i}^{t-1}P_{i}^{t}}$, while the second term represents the corresponding velocity deviation between them. This tracking process assumes that the trajectories of moving fingers are mostly smooth and plain, and thus their corresponding path coherences should reflect smooth motion in both directional and velocity deviations. The path coherence function can thus be derived and computed as:
  (11) $$\begin{array}{l} \varphi (P_{i}^{t-2},P_{i}^{t-1},P_{i}^{t})\\ \;\;\;\;\;\;\;\;\;\;\;\;\;\;\;\;=w_{1}(1-\text{cos }\theta )+w_{2}\left[1-2\left(\frac{\sqrt{d(P_{i}^{t-2},P_{i}^{t-1})\cdot d(P_{i}^{t-1},P_{i}^{t})}}{d(P_{i}^{t-2},P_{i}^{t-1})+d(P_{i}^{t-1},P_{i}^{t})}\right)\right]\\ \;\;\;\;\;\;\;\;\;\;\;\;\;\;\;\;=w_{1}\left(1-\frac{\bar{P_{i}^{t-2}P_{i}^{t-1}}\cdot \bar{P_{i}^{t-1}P_{i}^{t}}}{\left\|\bar{P_{i}^{t-2}P_{i}^{t-1}}\right\|\cdot \left\|\bar{P_{i}^{t-1},P_{i}^{t}}\right\|}\right)+w_{2}\left[1-2\left(\frac{\sqrt{\left\|\bar{P_{i}^{t-2}P_{i}^{t-1}}\right\|\cdot \left\|\bar{P_{i}^{t-1}P_{i}^{t}}\right\|}}{\left\|\bar{P_{i}^{t-2}P_{i}^{t-1}}\right\|+\left\|\bar{P_{i}^{t-1},P_{i}^{t}}\right\|}\right)\right] \end{array}$$
6. Hence, the path deviation of a given blob *P_i_* corresponding to its tracker ${\mathrm{TP}}_{i}^{t}$, denoted as $D_{i}({\mathrm{TP}}_{i}^{t})$, can be computed and obtained by:
  (12) $$D_{i}({\mathrm{TP}}_{i}^{t})=\sum_{t=2}^{n-1}d_{i}^{t}$$
7. Accordingly, when *m* blobs are detected within a time slot of image frames, the overall tracker deviation function **D** of the tracker sets of these *m* blobs can be obtained by:
  (13) $$D=\sum_{i=1}^{m}D_{i}$$

Based on the above-mentioned definitions utilized in the tracking process, the trackers formed by the detected touch blobs in the sequential images can be determined by minimizing the overall tracker deviation function **D** associated with these detected blobs. Accordingly, in each recursion of the tracking process for a incoming frame *t*, the newly detected touch blobs appearing in this incoming frame, denoted by ${\text{P}}^{t}=\{P_{i}^{t}|i=1,\ldots ,k^{\prime }\}$, will be analyzed and associated with the tracker sets of touch blobs that have already been tracked in the previous frames *t* − 1 and *t* − 2, denoted by ${\text{TP}}^{t-1}=\{{\mathrm{TP}}_{j}^{t-1}|j=1,\ldots ,k\}$ and ${\text{TP}}^{t-2}=\{{\mathrm{TP}}_{j^{\prime }}^{t-2}|j^{\prime }=1,\ldots ,k^{\prime }\}$, respectively. Thus, the current tracker set of the finger blobs **TP***^t^* can be updated by minimizing the overall tracker deviation function **D** associated with the sets of finger blobs in **P***^t^*, **TP***^t^*^−1^, and **TP***^t^*^−2^.

#### 3.2.2. Touch Event Identification and Occlusion Resolution Process

The proposed system can efficiently obtain trackers that reflect the spatial-temporal information of the detected finger blobs by applying the above-mentioned tracking process to the detected finger blobs. By analyzing the spatial-temporal information of the tracked finger blobs, the changing processes of the amount of finger blobs and the variation of the finger blob trajectories can reflect possible touching events of commands, or some occlusions of blob trackers caused by the blob extraction and tracking process. Such touch events may be: (1) When users press down or lift up their fingers, they change the number of finger blobs; (2) When users conduct a single-click or double-click action using their fingers, the corresponding finger blobs will quickly disappear and re-appear; (3) When users activate a zooming action by moving two fingers apart or close together along a straight line, the motion trajectories of a pair of finger blobs will form a straight line with increasing or decreasing distances; (4) Users may move two or more fingers close to each other for a while then split their fingers, or vice versa. In this case, the corresponding finger blobs might be occluded for a while during the blob extraction and tracking process. Using the trackers of the finger blobs ${\mathrm{TP}}_{i}^{t}\in {\text{TP}}^{t}$ obtained by the blob tracking process, the event analysis and occlusion resolution process can determine the corresponding touch events and resolve the occlusions of each finger blob tracker based on the spatial-temporal information of the tracked finger blobs.

To perform the touch event analysis and occlusion resolution process on the tracked finger blobs, the following spatial-temporal overlapping score of two finger blobs $P_{i}^{t}$ and $P_{j}^{t^{\prime }}$, detected at two different times *t* and *t’*, is defined as:

(14) $$S_{o}(P_{i}^{t},P_{j}^{t^{\prime }})=\frac{A(P_{i}^{t}\cap P_{j}^{t^{\prime }})}{Max(A(P_{i}^{t}),A(P_{j}^{t^{\prime }}))}$$

where $A(P_{i}^{t}\cap P_{j}^{t^{\prime }})$ represents the area of intersection of $P_{i}^{t}$ and $P_{j}^{t^{\prime }}$.

**Figure 7.**
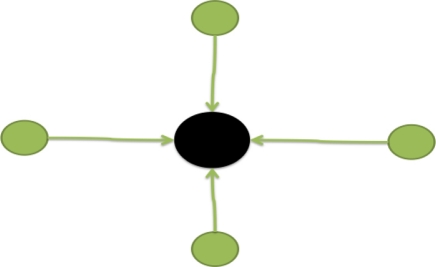
Illustration of a merging event.

**Figure 8.**
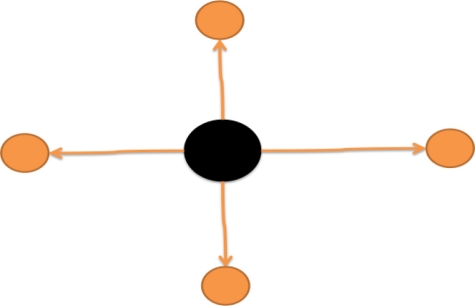
Illustration of a splitting event.

**Figure 9.**
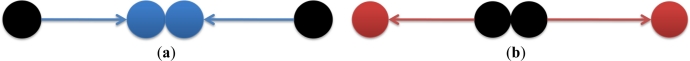
Illustrations of zooming action events. (**a**) Zooming out event. (**b**) Zooming in event.

While the finger blobs are being tracked, the touch event analysis and occlusion resolution process will determine the associated possible event states based on their spatial-temporal features. In the proposed system, the tracked finger blobs might be in one of seven possible event states, and the touch event analysis and occlusion resolution process will activate different touch events or operations according to the given event states of the tracked finger blobs in each given period. The proposed system uses the following tracking event states and associated actions for the tracked finger blobs:

1. Update: When the number of tracked finger blobs ${\mathrm{TP}}_{i}^{t}\in {\text{TP}}^{t}$ in the current frame is exactly equal to the number of those ${\mathrm{TP}}_{j}^{t-1}\in {\text{TP}}^{t-1}$ in the previous frame, then no touch event happens. In this case, the process simply updates the set of tracked finger blobs ${\mathrm{TP}}_{i}^{t}\in {\text{TP}}^{t}$ in the current frame with the ones ${\mathrm{TP}}_{j}^{t-1}\in {\text{TP}}^{t-1}$ in the previous frame according to the updated trackers obtained by the blob tracking process.
2. Appearing / Finger Pressing-down: If the newly appearing finger blobs $P_{i}^{t}\in P^{t}$ do not match any ${\mathrm{TP}}_{j}^{t-1}\in {\text{TP}}^{t-1}$ in the previous period, the user may have pressed additional fingers on the panel. In this case, new trackers are created for these finger blobs and appended to the updated set **TP***^t^* for the next recursion of the blob tracking process.
3. Disappearing/Finger Lifting-up: Some existing trackers of finger blobs ${\mathrm{TP}}_{j}^{t-1}\in {\text{TP}}^{t-1}$ do not match any newly detected finger blobs $P_{i}^{t}\in {\text{P}}^{t}$. This situation might occur when the user lifts up some fingers from the panel. A tracked finger blob may temporarily disappear or become occluded in some frames, and will soon re-appear in subsequent frames. This can happen when the user wants to activate a clicking command. To prevent these finger blobs from being regarded as newly-appearing finger blobs, the system retains their trackers through the next five frames to help identify possible clicking events. If a tracker of finger blob ${\mathrm{TP}}_{j}^{t-1}$ does not matched any finger blobs $P_{i}^{t}\in {\text{P}}^{t}$ for more than five succeeding frames, then this finger blob will be determined to have disappeared and its tracker will be removed from the tracker set **TP***^t^* in the following frames.
4. Clicking Action: If a tracked finger blob ${\mathrm{TP}}_{j}^{t-1}\in {\text{TP}}^{t-1}$ suddenly disappears for a short period (within five frames) and then quickly re-appears, or repeats this event again, in a hovering position within a specific area, the user may be performing a single or double clicking operation. Therefore, if any finger blobs $P_{i}^{t+k}\in {\text{P}}^{t+k}$ in the following short period of five frames (*i.e.*, *k* = 0,1,…,5) quickly re-appear, they may successfully match ${\mathrm{TP}}_{j}^{t-1}\in {\text{TP}}^{t-1}$ by the following matching condition:
  (15) $$S_{o}(P_{i}^{t+k},{\mathrm{TP}}_{j}^{t-1})>\tau_{m}$$
  To identify clicking events accurately, the threshold *τ_m_* should have reasonable spatial-temporal coherences for $P_{i}^{t+k}$ and ${\mathrm{TP}}_{j}^{t-1}$ to determine if they are associated with the same finger clicks. This study uses a value of *τ_m_* = 0.25 to obtain satisfactory click event identification results.
5. Merging: If a detected finger blob $P_{i}^{t}$ in the current frame matches multiple tracked finger blobs ${\mathrm{TP}}_{j}^{t-1}$, ${\mathrm{TP}}_{j+1}^{t-1}$ …, ${\mathrm{TP}}_{j+n}^{t-1}$ (Figure 7), this indicates that the user’s formerly separate fingers are currently touching. Therefore, the *matching condition* [Equation (15)] of the detected finger blob $P_{i}^{t}$ with the tracked finger blobs ${\mathrm{TP}}_{j}^{t-1}$, ${\mathrm{TP}}_{j+1}^{t-1}$ …, ${\mathrm{TP}}_{j+n}^{t-1}$ will be respectively checked, and any tracked finger blobs that satisfy the matching condition will be merged into a single tracker of the renewed ${\mathrm{TP}}_{i}^{t}$, and the tracker set **TP***^t^* will be updated.
6. Splitting: If one tracked finger blob ${\mathrm{TP}}_{j}^{t-1}$ in the previous frame is split into blobs $P_{i}^{t}$, $P_{i+1}^{t}$ …, $P_{i+m}^{t}$ in the current frame (Figure 8), then the user’s formerly closed fingers are becoming separated. Thus, the matching condition (Equation (15)) between this tracked finger blob ${\mathrm{TP}}_{j}^{t-1}\in {\text{TP}}^{t-1}$ is evaluated with the newly detected finger blobs $P_{i}^{t}$, $P_{i+1}^{t}$ …, $P_{i+m}^{t}$ that are possibly associated with the split fingers. If the newly detected blobs having the highest matching score *S_o_* with ${\mathrm{TP}}_{j}^{t-1}$ are still associated with the updated tracker ${\mathrm{TP}}_{j}^{t}$, the remaining newly detected blobs that match ${\mathrm{TP}}_{j}^{t-1}$ will be marked as having split from the same closed finger set and will begin their own new trackers ${\mathrm{TP}}_{j^{\prime }}^{t}$, ${\mathrm{TP}}_{j^{\prime }+1}^{t}$ …, ${\mathrm{TP}}_{j^{\prime }+m-1}^{t}$ in the updated tracker set **TP***^t^*.
7. Zooming Action: When a pair of two tracked finger blobs ${\mathrm{TP}}_{i}^{t}$ and ${\mathrm{TP}}_{j}^{t}$ move close together, or move from being close together to far apart along a straight line, this may indicate the user is zooming out or zooming in (as depicted in Figure 9(a) and Figure 9(b), respectively). When the trajectories of a pair of two distant finger blobs ${\mathrm{TP}}_{i}^{t}$ and ${\mathrm{TP}}_{j}^{t}$move close to each other along a straight line and eventually result in a *merging* event, then a *zooming out* event is identified. When a touched blob of two fingers starts with a *splitting* event into a pair of two blobs ${\mathrm{TP}}_{i}^{t}$ and ${\mathrm{TP}}_{j}^{t}$, and they move apart along a straight line, then a *zooming in* event is identified

After identifying the touch events based on the moving blobs in the video sequences captured from the proposed touch-sensing interface, the touch events are then be transformed into interactive commands for the corresponding computer applications, such as selecting, moving, clicking, or zooming some interactive objects on the display screen.

## 4. Experimental Results

**Figure 10.**
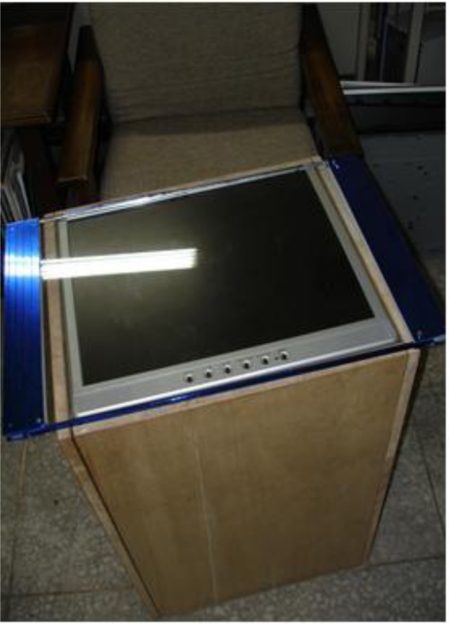
The prototype system of the proposed multi-touch sensing and display system.

This section presents several representative experiments for evaluating the performance of the proposed vision-based multi-touch finger detection, tracking, and event identification techniques using a prototype system of the proposed multi-touch sensing and display system (Figure 10).

The proposed vision-based multi-touch sensing and display techniques were implemented on a 2.4 GHz Pentium-IV personal computer platform. To provide better portability and maintainability for the proposed system, the software modules of the proposed vision-based techniques were implemented using C++ programming language with the Visual C++ 2008 development environment. By releasing the source code project associated with the software modules, the proposed vision-based techniques can be conveniently distributed and migrated onto different hardware platforms (such as ARM-based platforms) with various operating systems (such as Linux, Android, and Windows Mobile). Thus, the application developers can easily implement and design many customized multi-touch interactive application systems under various hardware platforms and software environments. The frame rate of the vision system is approximately 30 frames per second, and the resolution of each frame of captured image sequences is 640 pixels by 480 pixels per frame. An experimental set of several multi-touch sensing videos captured in various illumination conditions and application environments was adopted to evaluate the system’s finger detection and touch event identification. This experimental set consists of five experimental video sequences with a total of 16,841 captured frames and 1,113 desired interactive touch events in various conditions.

**Figure 11.**
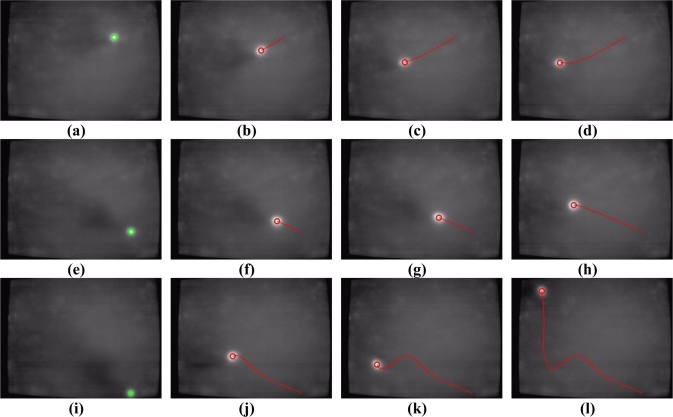
Touch finger detection and tracking results of the proposed system in Test sequence 1. **(a)** 19^th^ frame. **(b)** 26^th^ frame. **(c)** 30^th^ frame. **(d)** 33^rd^ frame. **(e)** 226^th^ frame. **(f)** 233^rd^ frame. **(g)** 235^th^ frame. **(h)** 243^rd^ frame. **(i)** 676^th^ frame. **(j)** 696^th^ frame. **(k)** 706^th^ frame. **(l)** 725^th^ frame.

**Figure 12.**
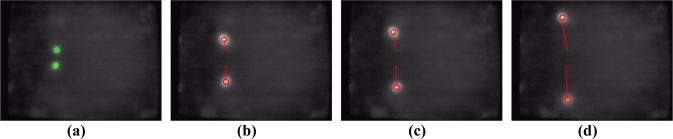

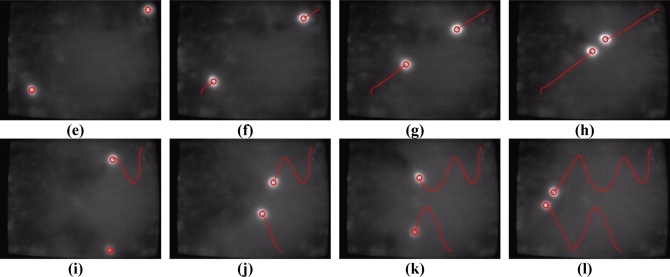
Touch finger detection and tracking results of the proposed system in Test sequence 2. **(a)** 187^th^ frame. **(b)** 193^rd^ frame. **(c)** 195^th^ frame. **(d)** 200^th^ frame. **(e)** 479^th^ frame. **(f)** 486^th^ frame. **(g)** 493^rd^ frame. **(h)** 503^rd^ frame. **(i)** 1607^th^ frame. **(j)** 1615^th^ frame. **(k)** 1625^th^ frame. **(l)** 1645^th^ frame.

**Figure 13.**
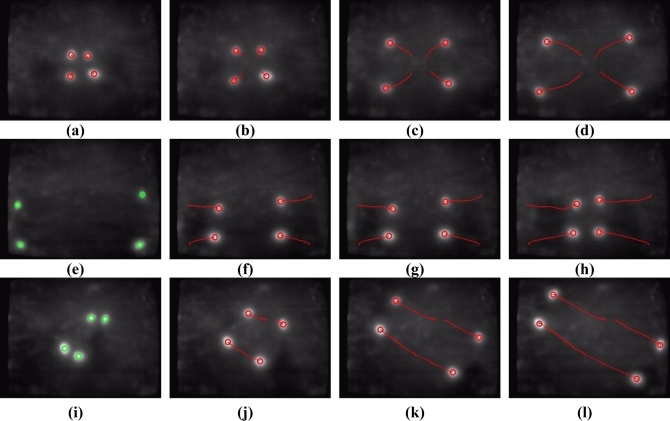
Touch finger detection and tracking results of the proposed system in Test sequence 3. **(a)** 33^rd^ frame. **(b)** 35^th^ frame. **(c)** 38^th^ frame. **(d)** 41^st^ frame. **(e)** 152^nd^ frame. **(f)** 164^th^ frame. **(g)** 166^th^ frame. **(h)** 174^th^ frame. **(i)** 2139^th^ frame. **(j)** 2148^th^ frame. **(k)** 2156^th^ frame. **(l)** 2170^th^ frame.

**Figure 14.**
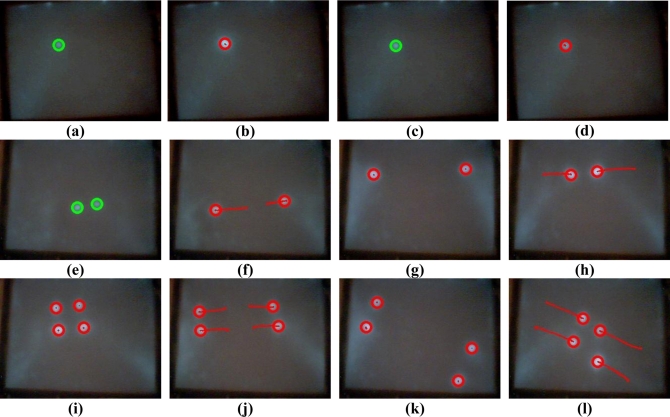
Touch finger detection and tracking results of the proposed system in Test sequence 4. **(a)** 168^th^ frame. **(b)** 170^th^ frame. **(c)** 174^th^ frame. **(d)** 178^th^ frame. **(e)** 216^th^ frame. **(f)** 267^th^ frame. **(g)** 476^th^ frame. **(h)** 535^th^ frame. **(i)** 861^st^ frame. **(j)** 902^nd^ frame. **(k)** 808^th^ frame. **(l)** 855^th^ frame.

**Figure 15.**
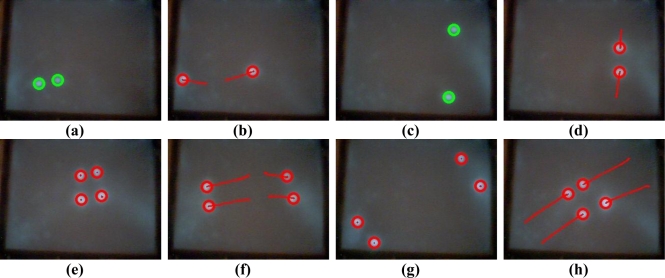

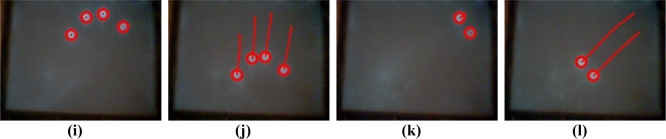
Touch finger detection and tracking results of the proposed system in Test sequence 5. **(a)** 427^th^ frame. **(b)** 475^th^ frame. **(c)** 3063^rd^ frame. **(d)** 3121^st^ frame. **(e)** 518^th^ frame. **(f)** 569^th^ frame. **(g)** 727^th^ frame. **(h)** 783^rd^ frame. **(i)** 785^th^ frame. **(j)** 858^th^ frame. **(k)** 221^st^ frame. **(l)** 307^th^ frame.

To evaluate the performance of the proposed vision-based multi-touch detection and tracking technique, Figures 11–15 exhibit the most representative experimental samples of touch sensing videos with different numbers of finger touches, various actions, and different illumination conditions. These figures indicate newly-detected touch fingers as green circles, while the tracked touch fingers are labeled as red circles, and their movements are drawn as red trajectories. Figures 11 and 12 show two usual multi-touch processing sequences with one or two touch finger at a time. The snapshots of the detection and tracking results in this figure show that the proposed vision-based technique correctly detected and tracked almost all of the moving finger touches.

The experimental samples in Figures 13–15 evaluate the detection and tracking results for more complicated multi-touch experimental scenes. The snapshots of the results in these figures show that although multiple touch fingers simultaneously move in different directions, perform various complex movements and actions, and sometimes these fingers move very close to each other, the proposed vision-based technique successfully detects and tracks most of finger movements.

This study adopts the Jaccard coefficient [39] as a detection score for the quantitative evaluation of touch finger detection performance. This score has been frequently used for evaluating performance in information retrieval. This measure is defined as:

(16) $$J=\frac{T_{p}}{T_{p}+F_{p}+F_{n}}$$

where *T_p_* (true positives) represents the number of correctly detected touch fingers, *F_p_* (false positives) represents the number of falsely detected touch fingers, and *F_n_* (false negatives) is the number of missed touch fingers. The Jaccard coefficient *J* for the touch finger detection results of each frame of the touch-sensing image sequences were determined by manually counting the number of correctly detected touch fingers, falsely detected touch fingers, and missed detections of touch fingers in each frame. The average value of the Jaccard coefficients *J* was then obtained from all frames of the captured video sequences using:

(17) $$\bar{J}=\sum_{N}J/N$$

where *N* is the total number of video frames. The ground-truth of detected touch fingers was acquired by manual counting. Table 1 shows the results of the quantitative evaluation of the proposed approach on touch finger detection.

This table clearly shows that the proposed approach provides high detection accuracy on touch fingers, and can thus provide efficient multi-touch sensing results that accurately reflect the user’s interactive commands.

In addition to evaluating the detection accuracy of the touch fingers of the proposed vision-based technique on single frames, this study evaluates the identification performance of touch events based on the spatial-temporal features of the finger blobs detected and tracked in consecutive image frames. Manually analyzing and discriminating the touch events appearing in the five test sequences shows that there are 1,113 discernible touch events of possible interactive gesture commands, including finger moving, clicking, and zooming action events (Table 2). These 1,113 touch events were adopted to evaluate the multi-touch event identification performance of the proposed vision-based technique. Table 2 reports the experimental data on multi-touch event identification performance, showing that the average event identification rate of the proposed vision-based technique is approximately 96.41%. This demonstrates that the proposed technique can provide highly accurate multi-touch event identification. Thus, interactive gesture commands can be correctly and appropriately generated for various multi-touch applications.

**Figure 16.**
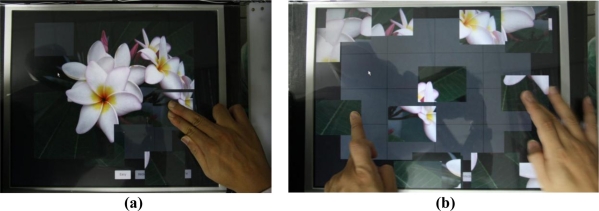
Example of performing the object selecting and moving commands on a puzzle game application using the proposed multi-touch sensing and display system. **(a)** Selecting objects. **(b)** Moving and rotating objects.

**Figure 17.**
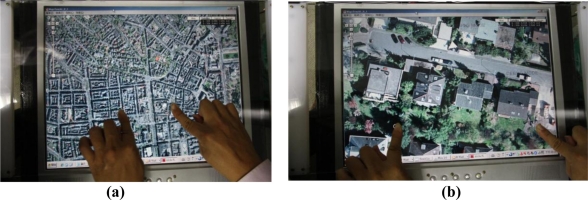
Example of performing the zooming in and zooming out commands on a Google Maps application using the proposed multi-touch sensing and display system. **(a)** Zooming out. **(b)** Zooming in.

The identified finger touch events can further be transformed into interactive commands for various applications. Figure 16 depicts an experimental scenario on running a puzzle game application using the proposed multi-touch sensing and display system. In this example, the user applied hands and fingers to select, move, or rotate objects (puzzle pieces) to complete a puzzle. Figure 17 shows another example of the application scenario using the Google Maps application on the proposed multi-touch sensing and display system. In this example, the user utilized two fingers to move and scale the map views, and the map views of the Google Maps are accordingly zoomed out and zoomed in [Figure 17(a) and Figure 17(b), respectively].

The computation time required to process one captured frame depends on the complexity of the touch command in the frame. Most of the computation time is spent on the connected-component analysis and touch blob tracking process. For an input video sequence with 640 × 480 pixels per frame, the proposed vision-based multi-touch sensing system requires an average of 12.2 milliseconds processing time per frame. This frugal computation cost ensures that the proposed system can effectively meet the requirements of real-time processing at over 80 frames per second. The above-mentioned experimental results of our numerous real tests on many different application scenarios and conditions on multi-touch sensing confirm that the proposed system can provide fast, real-time, and effective finger touch detection, tracking, and event identification for human-computer interactive applications. In our experimental analysis of the prototype system, the software modules of the proposed vision-based multi-touch sensing and display techniques only costs less than 5.0% of the CPU computational usage on average, and about 8,640 Kbytes of memory resource. These economic costs of computation and memory resources can efficiently provide the interactive multi-touch interfaces for many simultaneously executed software applications (such as the puzzle game and Google map applications demonstrated in Figure 16 and Figure 17). Thus, the proposed vision-based techniques can also be easily migrated and applied on embedded platforms for many consumer electronic products.

## 5. Conclusions

This study proposes a low-cost and efficient multi-touch sensing and display framework based on vision-based multi-touch finger detection, tracking, and event identification techniques and a low-cost hardware framework. The proposed multi-touch sensing and display system adopts a fast bright-blob segmentation process using automatic multilevel histogram thresholding to extract touch blob pixels formed by scattered infrared lights in the image sequences acquired by a video camera. This blob segmentation process approach is robust to the various ambient lighting conditions and spurious infrared noises that may appear in multi-touch sensing devices. The proposed system applies a connected-component analysis procedure to these segmented bright pixels to identify the connected components of touch finger blobs. The proposed blob tracking and event identification process analyzes the spatial-temporal information of these touch blobs in terms of consecutive captured frames, refines the detection results, and corrects errors and occlusions, and accordingly identifies possible touch events and actions. The proposed blob tracking and touch event identification process can efficiently determine meaningful touch events (such as finger moving, rotating, pressing, hovering, and clicking actions) by the analyzing spatial-temporal features of touch blobs in successive frames. The experiments in this study implement the proposed vision-based multi-touch finger detection, tracking, and event identification techniques on a low-cost hardware framework, forming a practical multi-touch sensing and display system. Experimental results in various application scenarios and operational conditions demonstrate that the proposed vision-based multi-touch sensing and display framework achieves fast, real-time, and effective finger touch detection, tracking, and event identification for many practical human-computer interactive applications and environments.

Regarding to the further researches on the vision-based multi-touch sensing and display systems, an extension to a large-scale and high-resolution multi-touch sensing and display system is desirable for extensive interactive applications. However, this extension will require much more computational costs and resources for achieving real-time operation performance on exploiting and processing large volumes of sensing video data, as well as the demand on guaranteeing high precision capabilities of the multi-touch finger detection, tracking, and event identification functions. In this aspect, further improvements and developments of the proposed system on the computational efficiency can be achieved by taking advantage of the computational power of the graphic processing units (GPU) [40,41]. Thus, the detection, tracking, and event identification processes of the large number of touch fingers and the associated large-scale sensing and display areas can be efficiently computed via the parallel processes executed on the GPUs.

## Figures and Tables

**Table 1.** Experimental data of the proposed approach on touch finger detection performance.

**Test sequence**	**No. of frames**	**No. of touch fingers appeared in each frame**	**Detection Score *J***

Test sequence 1	3,327	2,941	96.9%
Test sequence 2	3,330	5,662	96.2%
Test sequence 3	3,319	9,756	94.6%
Test sequence 4	3,510	6,879	98.7%
Test sequence 5	3,355	7,504	98.1%

Average Detection Score *J*	96.9%		

Total number of frames	16,841		

**Table 2.** Experimental data of the proposed technique on multi-touch event identification performance.

**Test sequence**	**No. of events**	**Event type**	**No. of individual events**	**No. of miss-detected events**	**Accuracy rate**
Test Sample 1	59	Move	59	2	96.6%
Test Sample 2	161	Move	83	4	95.1%
		Zoom in	41	2	95.1%
		Zoom out	37	1	97.3%
Test Sample 3	400	Move	306	13	95.8%
		Zoom in	42	2	95.2%
		Zoom out	52	2	96.1%
Test Sample 4	259	Move	66	2	96.9%
		Zoom in	54	2	96.3%
		Zoom out	54	2	96.3%
		Click	85	2	97.6%
Test Sample 5	234	Move	132	4	96.9%
		Zoom in	48	1	97.9%
		Zoom out	54	1	98.1%
Overall	1,113	1,113		40	96.41%
